# Activation of human α-carbonic anhydrase isoforms I, II, IV and VII with bis-histamine schiff bases and bis-spinaceamine substituted derivatives

**DOI:** 10.1080/14756366.2019.1630616

**Published:** 2019-06-25

**Authors:** Suleyman Akocak, Nabih Lolak, Silvia Bua, Alessio Nocentini, Claudiu T. Supuran

**Affiliations:** a Department of Pharmaceutical Chemistry, Faculty of Pharmacy, Adiyaman University, Adiyaman, Turkey;; b Dipartimento Neurofarba, Sezione di Scienze Farmaceutiche e Nutraceutiche, Università degli Studi di Firenze, Sesto Fiorentino, Italy

**Keywords:** Carbonic anhydrase, activator, histamine, spinaceamine, proton shuttle

## Abstract

A series of histamine bis-Schiff bases and bis-spinaceamine derivatives were synthesised and investigated as activators of four human (h) carbonic anhydrase (CA, EC 4.2.1.1) isoforms, the cytosolic hCA I, II and VII, and the membrane-associated hCA IV. All isoforms were effectively activated by the new derivatives, with activation constants in the range of 4.73–10.2 µM for hCA I, 6.15–42.1 µM for hCA II, 2.37–32.7 µM for hCA IV and 32 nM–18.7 µM for hCA VII, respectively. The nature of the spacer between the two histamine/spinaceamine units of these molecules was the main contributor to the diverse activating efficacy, with a very different fine tuning for the diverse isoforms. As CA activators recently emerged as interesting agents for enhancing cognition, in the management of CA deficiencies, or for therapy memory and artificial tissues engineering, our compounds may be considered as candidates for such applications.

## 1. Introduction

In previous research from our groups^1^^,^^2^, we reported two novel classes of activators of the enzyme carbonic anhydrase (CA, EC 4.2.1.1): the histamine Schiff bases^1^ and the spinaceamine derivatives^2^. As all CA activators (CAAs), these new classes of enzyme modulators also participate in the catalytic cycle of the enzyme^3–6^. Indeed, CAs are metalloenzymes, usually using Zn(II) ions within their active site for performing the efficient hydration of CO_2_ to bicarbonate and protons. A water molecule coordinated to the zinc ion becomes highly nucleophilic, and as hydroxide ion attacks the CO_2_ molecule bound within the active site of the enzyme, with formation of bicarbonate coordinated to the zinc (Equation (1))^7–10^. Another incoming water molecule subsequently displaces the bound bicarbonate, liberating it in solution, and leading to the formation of the acidic species of the enzyme, with water as zinc ligand Equation (1). In order to obtain the nucleophilic species of the enzyme, with the hydroxide coordinated to the zinc Equation (2), a proton transfer reaction must occur, which is the rate determining step of the entire catalytic cycle^3^^,^^7–10^.

$$H_{2}O$$

(1) $${\text{EZn}}^{2+}-{\text{OH}}^{-}+{\;\text{CO}}_{2}\Leftrightarrow {\text{EZn}}^{2+}-{\text{HCO}}_{3}{}^{-}\Leftrightarrow {\text{EZn}}^{2+}-{\text{OH}}_{2}+{\;\text{HCO}}_{3}{}^{-}$$

(2) $${\text{EZn}}^{2+}-{\text{OH}}_{2}\Leftrightarrow {\text{EZn}}^{2+}-{\text{HO}}^{-}+{\;H}^{+}$$

It has been demonstrated that the activators A in Equation (3) intervene in this step, providing an alternative pathway for the release of protons and formation of the zinc hydroxide species of the enzyme^3–6^.

(3) $${\text{EZn}}^{2+}-{\text{OH}}_{2}+\;A\Leftrightarrow [{\text{EZn}}^{2+}-{\text{OH}}_{2}-\;A]\Leftrightarrow [{\text{EZn}}^{2+}-{\text{HO}}^{-}-{\;\text{AH}}^{+}]\Leftrightarrow {\text{EZn}}^{2+}-{\text{HO}}^{-}+{\;\text{AH}}^{+}$$

enzyme − activator complexes

The activator molecule participates to the rate-determining step of the catalytic cycle, that is, the proton shuttling between the zinc-coordinated water molecule and the environment, with the formation of the zinc hydroxide species of the enzyme^3–6^^,^^11^. In many CA isoforms, it has been shown that residue His64 placed in the middle of the active site cavity is involved in this phenomenon, acting as a natural proton shuttle residue during the catalytic cycle^11^. Confirmation that CAAs have a similar role to His64, that is, shuttling of the protons from the active site to the environment and facilitation of the formation of the nucleophilic enzyme species, came from many X-ray crystallographic studies of isoforms CA I and II complexed with amine and amino acid activators^3–6^. Histamine, the first CAA investigated by means of X-ray crystallography^3^, was observed to bind at the entrance of the active site cavity, distant from the zinc ion, and participating in a network of hydrogen bonds involving several water molecules, which, as for His64, favour the release of the proton from the water molecule coordinated to the zinc, to the reaction medium^3^. X-ray crystal of other CAAs reinforced the above findings: all activators bind in the same active site region, at the entrance of the cavity, from where they can enhance the formation of the zinc hydroxide species of the enzyme, by favouring the proton shuttling between the cavity and the reaction medium^3–6^. Furthermore, recently, it has also been shown the CAAs may have pharmacological applications for enhancing cognition, in the management of CA deficiencies, for therapy memory and for obtaining artificial tissues^12^. Thus, there is a strong interest in designing CAAs belonging to various chemical classes, in order to detect compounds with high efficacy and eventually isoform-selective action, considering that in humans at least 15 CA isoforms were described so far^7^. Here, we report some new CAAs obtained by considering our previous findings, that is, histamine Schiff bases and spinaceamine derivatives, which posses efficient CA activating properties^1^^,^^2^.

## 2. Materials and methods

### 2.1. Chemistry

All chemicals and anhydrous solvents were purchased from Sigma-Aldrich, Merck, Alfa Aesar and TCI and used without further purification. Melting points (mp) were determined with SMP30 melting point apparatus in open capillaries and are uncorrected. FT-IR spectra were recorded by using Perkin Elmer Spectrum 100 FT-IR spectrometer. Nuclear Magnetic Resonance (^1^H-NMR and ^13 ^C-NMR) spectra of compounds were recorded using a Bruker Advance III 300 MHz spectrometer in DMSO-d_6_ and TMS as an internal standard operating at 300 MHz for ^1^H-NMR and 75 MHz for ^13 ^C-NMR. Thin layer chromatography (TLC) was carried out on Merck silica gel 60 F_254_ plates.

#### 2.1.1. General procedure for the synthesis of bis-histamine schiff bases H1, H2, H3 and H4

Potassium hydroxide (10 mmol) was added to a stirred suspension of histamine dihydrochloride (5 mmol) in dry EtOH (10–15 ml) at room temperature. After stirring for 2 h, the precipitate salt (KCl) was filtered off and the filtrate was treated with a solution of bis-aldehydes (2.5 mmol) in dry EtOH (20–25 ml). The homogeneous mixture was stirred overnight at room temperature. The completion of the reaction was monitored by TLC and FT-IR. The excess solvent was evaporated and the oily residue was crystallized with ethyl acetate and diethylether to obtain the corresponding bis-histamine Schiff base derivatives. The desired final products **H1, H2, H3,** and **H4** were dried under vacuum and fully characterised by FT-IR, ^1^H-NMR, ^13 ^C-NMR, elemental analysis and melting points.

**1,4-Phenylenebis(methanylylidene))bis(2-(1H-imidazol-4-yl)ethanamine) (H1):** Yield: 65%; Colour: cream powder, mp: 165–168^0^ C; FT-IR (cm^−1^): 3085, 3020, 2841, 2631, 1636 (-C = N-), 1453, 1292, 821; ^1^H-NMR (DMSO-d_6_, 300 MHz, δ ppm): 8.68 (*s*, 2H, -N=CH-), 8.08 (d, 2H, *J* = 2.4, H-2 Im), 7.98 (d, 4H, *J* = 7.5, Ar-H), 7.55 (*s*, 2H, H-5 Im), 3.75 (*t*, 4H, *J* = 5.5, -CH_2_CH_2_-Im), 2.95 (*t*, 4H, *J* = 5.5, -CH_2_CH_2_-Im): ^13 ^C-NMR (DMSO-d_6_, 75 MHz, δ ppm): 167.5 (-N = CH-), 135.4, 133.2, 131.7, 130.4, 116.3, 55.5, 29.8;

**1,3-Phenylenebis(methanylylidene))bis(2-(1H-imidazol-4-yl)ethanamine) (H2):** Yield: 48%; Colour: cream powder, mp: 210-212^0^ C; FT-IR (cm^−1^): 3120, 3024, 2922, 2851, 1615 (-C = N-), 1437, 1290, 822; ^1^H-NMR (DMSO-d_6_, 300 MHz, δ ppm): 8.70 (*s*, 2H, -N=CH-), 8.25 (*s*, 1H, Ar-H), 8.12 (d, 2H, *J* = 2.8, H-2 Im), 8.00 (d, 2H, *J* = 7.2, Ar-H), 7.62 (*s*, 2H, H-5 Im), 7.58 (*m*, 1H, Ar-H), 3.78 (*t*, 4H, *J* = 5.8, -CH_2_CH_2_-Im), 2.98 (*t*, 4H, *J* = 5.8, -CH_2_CH_2_-Im): ^13 ^C-NMR (DMSO-d_6_, 75 MHz, δ ppm): 167.2 (-N = CH-), 138.6, 136.2, 133.6, 131.2, 130.8, 128.6, 116.6, 55.8, 29.9;

**1,2-Phenylenebis(methanylylidene))bis(2-(1H-imidazol-4-yl)ethanamine)(H3):** Yield: 35%; Colour: cream powder, mp: 222–225^0^ C; FT-IR (cm^−1^): 3117, 2926, 2849, 1648 (-C = N-), 1434, 1223, 828; ^1^H-NMR (DMSO-d_6_, 300 MHz, δ ppm): 8.71 (*s*, 2H, -N=CH-), 8.15 (d, 2H, *J* = 6.9, Ar-H), 8.10 (d, 2H, *J* = 2.8, H-2 Im), 7.60 (*s*, 2H, H-5 Im), 7.48 (*m*, 2H, Ar-H), 3.76 (*t*, 4H, *J* = 5.5, -CH_2_CH_2_-Im), 2.97 (*t*, 4H, *J* = 5.5, -CH_2_CH_2_-Im): ^13 ^C-NMR (DMSO-d_6_, 75 MHz, δ ppm): 166.8 (-N = CH-), 138.4, 135.8, 132.9, 131.7, 130.4, 128.3, 116.9, 55.9, 29.7;

**Furan-2,5-diylbis(methanylylidene))bis(2-(1H-imidazol-4-yl)ethanamine) (H4):** Yield: 32%; Colour: brown powder, mp: 114-117^0^ C; FT-IR (cm^−1^): 3107, 3016, 2926, 2853, 1621 (-C = N-), 1433, 1224, 816; ^1^H-NMR (DMSO-d_6_, 300 MHz, δ ppm): 8.88 (*s*, 2H, -N=CH-), 7.99 (d, 2H, *J* = 2.2, H-2 Im), 7.74 (*s*, 2H, H-5 Im), 6.85 (d, 2H, *J* = 2.8, furan), 3.52 (*t*, 4H, *J* = 5.8, -CH_2_CH_2_-Im), 2.88 (*t*, 4H, *J* = 5.8, -CH_2_CH_2_-Im): ^13 ^C-NMR (DMSO-d_6_, 75 MHz, δ ppm): 166.2 (-N = CH-), 151.4, 144.9, 138.3, 134.7, 119.5, 113.2, 56.3, 31.5;

#### 2.1.2. General procedure for the synthesis of bis-Spinaceamine substituted compounds SPH1, SPH2, and SPH4

To a solution of 5 mmol of histamine dihydrochloride in 10 ml of water were added solutions of 15 mmol of sodium hydroxide (NaOH) and 2.5 mmol of appropriate bis-aldehyde derivatives in 15 ml of ethanol. The reaction mixture was heated overnight at 80 °C. The completion of the reaction was monitored by TLC and FT-IR. After that, the mixture was allowed to cool to room temperature and the formed precipitate was filtered off. The crude powders were recrystallized from hot water and dried under vacuum at 40 °C to afford **SPH1, SPH2,** and **SPH4** which were fully characterized by FT-IR, ^1^H-NMR, ^13 ^C-NMR, and melting points.

**1,4-Bis(4,5,6,7-tetrahydro-3H-imidazo[4,5-c]pyridin-4-yl)benzene (SPH1):** Yield: 56%; Colour: cream powder, mp: 95-98^0^ C; FT-IR (cm^−1^): 3122, 3024, 2956, 2971, 2926, 1606, 1455, 963, 817; ^1^H-NMR (CD_3_OD, 300 MHz, δ ppm): 7.78 (*s*, 2H, H-2 Im), 7.46 (d, 4H, Ar-H), 5.10 (*s*, 2H, -CH-), 3.35 (*m*, 4H,-CH_2_CH_2_-Im), 2.92 (*t*, 4H, *J* = 5.2, -CH_2_CH_2_-Im): ^13 ^C-NMR (CD_3_OD, 75 MHz, δ ppm): 141.7, 135.9, 134.2, 129.8, 128.1, 127.3, 113.9, 64.2, 39.5, 30.0;

**1,3-Bis(4,5,6,7-tetrahydro-3H-imidazo[4,5-c]pyridin-4-yl)benzene (SPH2):** Yield: 45%; Colour: white powder, mp: 190-192^0^ C; FT-IR (cm^−1^): 3117, 3036, 2922, 2852, 1611, 1448, 947, 817; ^1^H-NMR (CD_3_OD, 300 MHz, δ ppm): 7.81 (*s*, 2H, H-2 Im), 7.52 (*m*, 1H, Ar-H), 7.22 (d, 2H, Ar-H), 7.05 (*s*, 1H, Ar-H), 5.08 (*s*, 2H, -CH-), 3.33 (*m*, 4H,-CH_2_CH_2_-Im), 2.93 (*t*, 4H, *J* = 5.5, -CH_2_CH_2_-Im): ^13 ^C-NMR (CD_3_OD, 75 MHz, δ ppm): 141.2, 136.5, 134.1, 129.4, 127.9, 127.7, 113.5, 64.4, 39.3, 29.7;

**2,5-Bis(4,5,6,7-tetrahydro-3H-imidazo[4,5-c]pyridin-4-yl)furan (SPH4):** Yield: 48%; Colour: orange mp: 185-188^0^ C; FT-IR (cm^−1^): 3012, 2919, 2849, 1608, 1379, 1322, 1135, 1011, 813; ^1^H-NMR (CD_3_OD, 300 MHz, δ ppm): 7.92 (*s*, 2H, H-2 Im), 6.65 (d, 2H, *J* = 5.5, furyl), 5.25 (*s*, 2H, -CH-), 3.36 (*m*, 4H,-CH_2_CH_2_-Im), 2.97 (*t*, 4H, *J* = 5.8, -CH_2_CH_2_-Im): ^13 ^C-NMR (CD_3_OD, 75 MHz, δ ppm): 151.9, 143.4, 139.8, 134.7, 118.6, 114.2, 106.8, 63.2, 39.4, 29.2.

### 2.2. CA activation

An Sx.18Mv-R Applied Photophysics (Oxford, UK) stopped-flow instrument has been used to assay the catalytic activity of various CA isozymes for CO_2_ hydration reaction^13^. Phenol red (at a concentration of 0.2 mM) was used as indicator, working at the absorbance maximum of 557 nm, with 10 mM Hepes (pH 7.5) as buffer, 0.1 M NaClO_4_ (for maintaining constant ionic strength), following the CA-catalysed CO_2_ hydration reaction for a period of 10 s at 25 °C. The CO_2_ concentrations ranged from 1.7 to 17 mM for the determination of the kinetic parameters and inhibition constants. For each activator at least six traces of the initial 5–10% of the reaction have been used for determining the initial velocity. The uncatalysed rates were determined in the same manner and subtracted from the total observed rates. Stock solutions of activators (at 0.1 mM) were prepared in distilled-deionised water and dilutions up to 1 nM were made thereafter with the assay buffer. Enzyme and activator solutions were pre-incubated together for 15 min prior to assay, in order to allow for the formation of the enzyme–activator complexes. The activation constant (K_A_), defined similarly with the inhibition constant K_I_, can be obtained by considering the classical Michaelis–Menten equation (Equation (4), which has been fitted by non-linear least squares by using PRISM 3:

(4) $$v\;=v_{\text{max}}/\left\{1+\left(K_{M}/\left[S\right]\right)\left(1+{\left[A\right]}_{f}/K_{A}\right)\right\}$$

where [A]_f_ is the free concentration of activator.

Working at substrate concentrations considerably lower than K_M_ ([S] ≪K_M_), and considering that [A]_f_ can be represented in the form of the total concentration of the enzyme ([E]_t_)and activator ([A]_t_), the obtained competitive steady-state equation for determining the activation constant is given by Equation (5):

(5) $$v=v_{0}.K_{A}/\left\{K_{A}+{\left({\left[A\right]}_{t}-0.5\{\left({\left[A\right]}_{t}+{\left[E\right]}_{t}+K_{A}\right)-{\left({\left[A\right]}_{t}+{\left[E\right]}_{t}+K_{A}\right)}^{2}-4{\left[A\right]}_{t}.{\left[E\right]}_{t}\right)}^{1/2}\right\}\}$$

where v_0_ represents the initial velocity of the enzyme-catalyzed reaction in the absence of activator^14–18^. All CAs were recombinant proteins obtained as reported earlier^18^.

## 3. Results and discussion

### 3.1. Chemistry

**Scheme 1.**
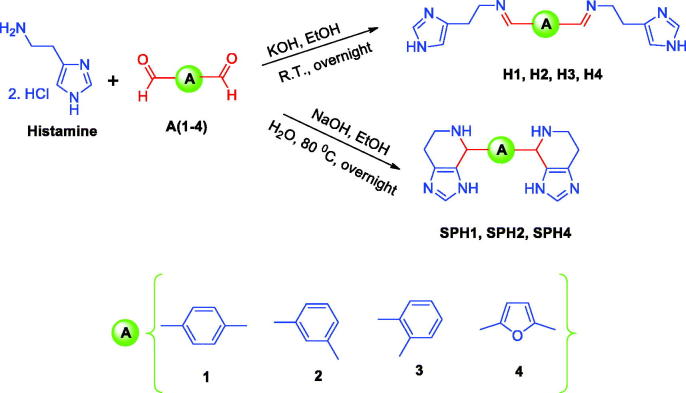
General synthetic route for the synthesis of the bis-histamine Schiff bases and bis-spinaceamine substituted compounds (incorporating the fused imidazole ring system).

The rationale for designing CAAs presented in this work is based on our previous data which showed efficient CA VII activating effects for derivatised histamine Schiff base compounds and spinaceamine derivatives^1^^,^^2^. Therefore, in this work, a number of structurally diverse bis-histamine Schiff bases and bis-Spinaceamine substituted compounds(ring-closure product of histamine Schiff bases) were synthesised according to general synthetic routes as illustrated in Scheme 1. In order to generate chemical diversity, different bis-aldehydes were chosen, possessing aromatic and heterocyclic moieties, and they were reacted with histamine leading to the bis-histamine Schiff bases and bis-Spinaceamine substituted compounds **SH1, H2, H3, H4, SPH1, SPH2, and SPH4** (Scheme 1). All the synthesised compounds were fully characterised by using several analytical and spectral data (see experimental part for details).

In the current work, the synthesis of a series of bis-histamine Schiff bases and bis-spinaceamine substituted compounds was carried out with some modifications of the literature procedures^2^. Briefly, histamine dihydrochloride was coupled with substituted aromatic and heterocyclic bis-aldehydes, leading to the formation of of bis-histamine Schiff bases and ring-closure products of histamine. The structures of bis-histamine Schiff bases and bis-spinaceamine substituted compounds were confirmed by using several analytical and spectral data (FT-IR, ^1^H-NMR, ^13 ^C-NMR, and melting points) as described in the experimental part.

### 3.2. CA activation

Considering the fact that the new heterocyclic derivatives **H1-H4** and **SPH1, 2** and**4** reported here incorporate in their molecules two functionalities with a pKa appropriate for acting as proton shuttles in the CA catalytic cycle^3^^,^^12^, we have investigated them as CAAs against the following four CA isoforms with important physiological functions: the three cytosolic enzymes (h = human), hCA I, II and VII^19^, and the membrane-associated hCA IV^20^. They are involved in various pathologies, both in the CNS, kidneys, eyes and other organs in which they are highly abundant^21–23^.

The following structure–activity relationship (SAR) can be evidenced from data of Table 1:

**Table 1.** CA activation data with bis-histamines **H1-H4** and bis-spinaceamines **SPH(1, 2** and**4)** and histamine(HST) as a standard activator, by a stopped-flow CO_2_ hydrase assay^13^.

	K_A_ (µM)^a^			
Compound	hCA I	hCA II	hCA IV	hCA VII
**H1**	4.73	42.1	3.96	9.02
**H2**	6.15	30.7	3.28	18.7
**H3**	18.4	25.9	10.9	21.3
**H4**	7.13	20.3	3.45	0.085
**SPH1**	10.2	8.21	32.7	0.032
**SPH2**	6.29	6.15	8.12	0.039
**SPH4**	9.87	19.2	2.37	0.035
**HST**	2.10	125	4.03	37.6

a Mean from 3 different determinations (errors in the range of 5–10% of the reported values, data not shown).

- Among the four investigated isoforms, hCA VII was the most sensitive to these activators (similar to the lead compounds used for obtaining these derivatives^1^^,^^2^), followed by hCA IV and I, whereas hCA II was the least sensitive to the activating effects of these compounds. However, these new derivatives reported here – **H1-H4** and **SPH(1, 2** and**4)** – were much more effective as hCA II activators compared to histamine (HST), a standard activator^3^ (Table 1).
- The slow cytosolic isoform hCA I was activated efficiently by **H1-H4** and **SPH(1, 2** and**4),** with K_A_s ranging between 4.73–18.4 µM. Similar activities were observed for the bis-histamine Schiff bases and the bis-spinaceamine derivatives, with the main factor influencing activity being the spacer between the two imidazole moieties. Indeed, for this isoform, the *p*-phenylene spacer present in **H1** and the *m*-phenylene one, present in **H2** and **SPH2**, led to the most effective activators (Table 1):
- The fast cytosolic enzyme hCA II was also effectively activated by the new derivatives, with K_A_s ranging between 6.15 and42.1 µM (compared to a K_A_ of 125 µM for histamine). The rationale of our drug design was in fact to introduce two proton shuttling moieties, of the histamine/spinaceamine type, in order to enhance the affinity for the enzyme and to facilitate the rate-determining step of the catalytic cycle. Although for hCA I this is not obvious, for hCA II the activating effects of the bis-derivatives investigated here are indeed much higher compared to the mono-derivatives incorporating just one proton shuttling moiety, as histamine. In fact, the best bis-activator of hCA II, compound **SPH2**, is 20.3-times a more effective activator compared to histamine (Table 1).
- (iv)The membrane-anchored hCA IV was activated by the new derivatives with activation constants ranging between 2.37 and 32.7 µM. Many of the new activators (e.g., **H1, H2, H4** and **SPH4**) were more effective than histamine (K_A_ of 4.03 µM) whereas the remaining ones were slightly less effective. Again the spacer between the two imidazole(-like) units was the main factor responsible of these effects, with the 2,5-furylene one leading to effective hCA IV activators (**H4** and **SPH4**).
- The most activatable isoform was the brain-associated hCA VII, for which the new activators reported here showed K_A_s ranging between 32 nM and 18.7 µM. One histamine bis-Schiff base (**H4**) and all three bis-spinaceamines **SPH1, 2** and **4**, were nanomolar hCA VII activators, with affinities of 32-85 nM (Table 1). Thus, for these last derivatives, the nature of the spacer had less influence on activity, as all of them show a behavior of potent activators, whereas for the histamine derivatives only the furyl-containing compound (**H4**) was an effective activator, with the phenylene ones **H1-H3** being several orders of magnitude less effective.

## 4. Conclusions

We report here a small series of histamine bis-Schiff bases and bis-spinaceamine derivatives, which were synthesized by original procedures and investigated as activators of four hCA isoforms involved in a variety of diseases, the cytosolic hCA I, II and VII, and the membrane-associated hCA IV. All these isoforms were effectively activated by the new derivatives, with activation constants in the range of 4.73–10.2 µM for hCA I, 6.15–42.1 µM for hCA II, 2.37–32.7 µM for hCA IV and 32 nM -18.7 µM for hCA VII, respectively. The nature of the spacer between the two histamine/spinaceamine units of these molecules was the main contributor to the diverse activating efficacy, with a very different fine tuning for the diverse isoforms. As CA activators recently emerged as interesting agents for enhancing cognition, in the management of CA deficiencies, or for therapy memory and artificial tissues engineering, our compounds may be considered as candidates for such applications.
